# Supplementary material for: Unleashing the Influence of cAMP Receptor Protein: The Master Switch of Bacteriocin Export in Pectobacterium carotovorum subsp. carotovorum
Source: Int J Mol Sci. 2023 Jun 5;24(11):9752. doi: 10.3390/ijms24119752 (PMC10253576; doi:10.3390/ijms24119752)
Supplement: Supplementary file 1 [file ijms-24-09752-s001.zip › ijms-2394330-supplementary.pdf]

Supplementary Table S1. Primers used in this study.

Primer	Sequence (5′-3′)
CRP-forward-primer	AGGAG GATCC CGAAT GGTTC
CRP-reverse-primer	GCGAC AAAGC TTTAG CGAG
CRP-sens1	AGGAGGATCCCGAATGGTTC
CRP-antisens1	GCGACAAAGCTTTAGCGAG
Biotin-CAP site 1	GATATTTAAAATCAGTTTATTAACTGTGTT (5'-Biotin)
complement-CAP site 1	AACACAGTTAATAAACTGATTTTAAATATC
Biotin-CAP site 2	ATACAGACATGGCATTTTTTGATGGAGAAC (5'-Biotin)
complement-CAP site 2	GTTCTCCATCAAAAAATGCCATGTCTGTAT
caro-S3K-forward	ATGAT TAAGT ACCGT TTATA TGCTC
caro-S3K-reverse	TCATT GCGAC TCCCT CATAT
PCC_DGC_F_upstream	CTCAC TGTTG CTGAC ATGC
PCC_DGC_R_downstream	ATTCA GGCAA CTTCG GTTC
16s-forward	CTGGA CAAAG ACTGA CGCTC
16s-reverse	CGCTG GCAAC AAAGG ATAAG
